# Natural mucosal barriers and COVID-19 in children

**DOI:** 10.1172/jci.insight.148694

**Published:** 2021-05-10

**Authors:** Carl A. Pierce, Sharlene Sy, Benjamin Galen, Doctor Y. Goldstein, Erika Orner, Marla J. Keller, Kevan C. Herold, Betsy C. Herold

**Affiliations:** 1 Department of Microbiology and Immunology, Albert Einstein College of Medicine, Bronx, New York, USA.; 2 Department of Pediatrics, the Children’s Hospital at Montefiore and Albert Einstein College of Medicine, Bronx, New York, USA.; 3 Department of Medicine and; 4 Department of Pathology, Montefiore Medical Center and Albert Einstein College of Medicine, Bronx, New York, USA.; 5 Departments of Immunobiology and Internal Medicine, Yale University, New Haven, Connecticut, USA.

**Keywords:** Infectious disease, Innate immunity

## Abstract

**BACKGROUND:**

Coronavirus disease 2019 (COVID-19) is more benign in children compared with adults for unknown reasons. This contrasts with other respiratory viruses where disease manifestations are often more severe in children. We hypothesize that a more robust early innate immune response to SARS coronavirus 2 (SARS-CoV-2) protects against severe disease.

**METHODS:**

Clinical outcomes, SARS-CoV-2 viral copies, and cellular gene expression were compared in nasopharyngeal swabs obtained at the time of presentation to the emergency department from 12 children and 27 adults using bulk RNA sequencing and quantitative reverse-transcription PCR. Total protein, cytokines, and anti–SARS-CoV-2 IgG and IgA were quantified in nasal fluid.

**RESULTS:**

SARS-CoV-2 copies, angiotensin-converting enzyme 2, and TMPRSS2 gene expression were similar in children and adults, but children displayed higher expression of genes associated with IFN signaling, NLRP3 inflammasome, and other innate pathways. Higher levels of IFN-α2, IFN-γ, IP-10, IL-8, and IL-1β protein were detected in nasal fluid in children versus adults. Children also expressed higher levels of genes associated with immune cells, whereas expression of those associated with epithelial cells did not differ in children versus adults. Anti–SARS-CoV-2 IgA and IgG were detected at similar levels in nasal fluid from both groups. None of the children required supplemental oxygen, whereas 7 adults did (*P* = 0.03); 4 adults died.

**CONCLUSION:**

These findings provide direct evidence of a more vigorous early mucosal immune response in children compared with adults and suggest that this contributes to favorable clinical outcomes.

**FUNDING:**

NIH grants R01 AI134367, UL1 TR002556, T32 AI007501, T32GM007288, P30 AI124414; an Albert Einstein College of Medicine Dean’s COVID-19 Pilot Research Award; and the Eric J. Heyer, MD, PhD Translational Research Pilot Project Award.

## Graphical abstract

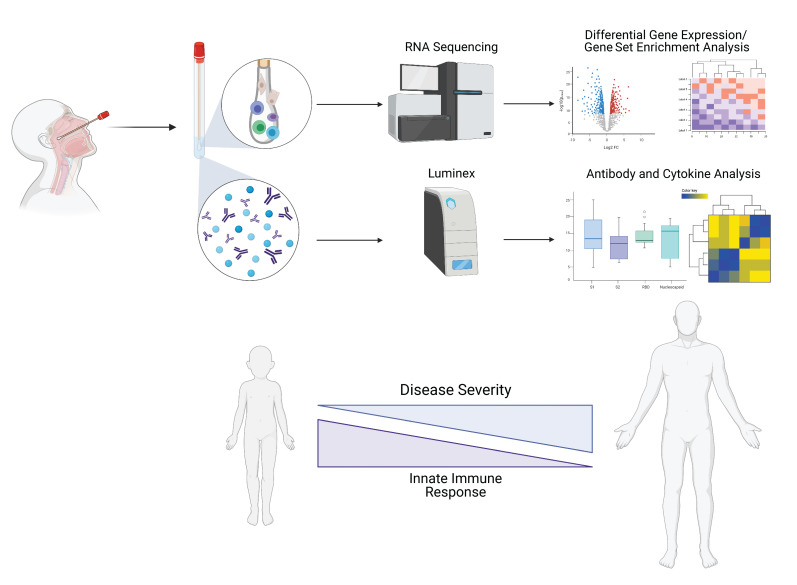

## Introduction

Epidemiological studies have consistently shown that children infected with SARS coronavirus 2 (SARS-CoV-2) have a milder clinical course with significantly less morbidity and mortality than adults. The CDC estimates that approximately 1.2%–3.3% of total hospitalizations and less than 0.21% of deaths from coronavirus disease 2019 (COVID-19) are in children (1). This experience is in contrast to other respiratory viruses, such as influenza or respiratory syncytial virus, where disease manifestations in children are often more severe than adults (2). Several hypotheses have been proposed to explain why children are protected from more severe outcomes with COVID-19, including differences in expression of angiotensin-converting enzyme 2 (ACE2), the receptor for viral entry, resulting in lower viral loads; presence of antibodies to common cold coronaviruses that might provide partial protection; and a more robust innate response early in the course of infection that mitigates against a vigorous adaptive response (3, 4). However, recent studies have shown that ACE2 expression is not reduced in children and may actually be lower in adults (5). Surveys of children infected with COVID-19 have found that the amount of SARS-CoV-2 RNA detected in nasopharyngeal (NP) swabs is at least as high in children compared with adults (6). It is also unlikely that antibodies that are cross-reactive to other viruses explain the clinical differences, given that we previously found that antibody levels to other common cold human coronaviruses (229E, NL63, HKU1) were similar in adults and children (7). In addition, although common cold coronavirus antibody levels may be boosted in response to SARS-CoV-2 infection, they do not provide protection (7, 8).

In a previous analysis of patients hospitalized with COVID-19, we found that serum levels of IL-17A and IFN-γ collected early in the course of disease were higher in children versus adults and correlated significantly and inversely with age (7). The source of these cytokines did not appear to be PBMCs because adults had more robust T cell responses to the spike protein. These findings suggested that other cells, such as innate immune cells within the respiratory tract, might be the source of the IL-17A and IFN-γ and that a more vigorous mucosal innate response could account for the milder clinical course in children. To test the hypothesis that age-related outcomes reflect differences in host responses at the site of viral exposure, we collected NP swabs from pediatric and adult patients who presented to the emergency department at Montefiore Medical Center in the Bronx, New York; analyzed the harvested cells for gene expression; and measured the levels of cytokines and antibodies in the mucosa.

## Results

**Figure 1:**
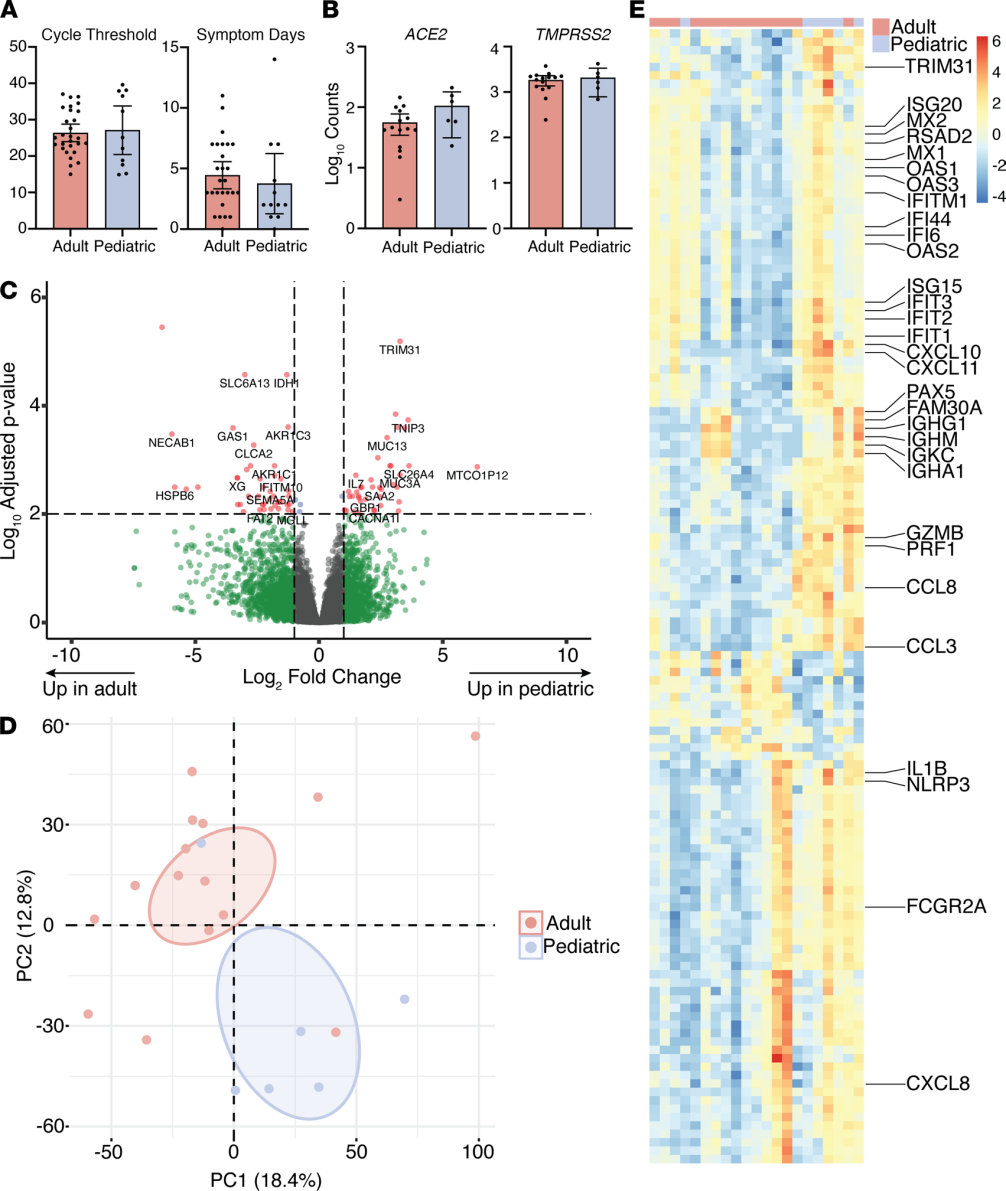
Transcriptional profiles in pediatric and adult nasopharyngeal samples. (**A**) Ct values and days of symptoms prior to presentation in 27 adult and 11 pediatric patients, and *ACE2* read counts in cells isolated from NP swabs obtained at presentation in 15 adult and 6 pediatric samples. Bars show mean ± 95% CI. (**B**) The expression of *ACE2* and *TMPRSS2* from the RNA-Seq studies. (**C**) Volcano plot of the expression of genes analyzed by RNA-Seq more strongly in pediatric (right) and adult (left) patients. (**D**) Principal component plot of RNA-Seq data; *n* = 15 adult and 6 pediatric samples. Ovals are 95% confidence ellipses. (**E**) Heatmap showing expression of the top 50 contributing genes in principal components 1, 2, and 3.

The demographics and clinical characteristics of the study population are described in Table 1. The emergency department visits were prompted by COVID-19 symptoms in 9/12 pediatric (fever and cough) and 25/27 adult (fever, cough, or dyspnea) patients, and the number of symptom days prior to presentation did not differ significantly between the groups (Figure 1A). In addition, the levels of SARS-CoV-2 viral RNA (quantified by Ct values) in NP swabs were similar when comparing the pediatric and adult patients (Figure 1A). However, the outcomes differed. Adults were more likely to be admitted to the hospital (81% vs. 42%, *P* = 0.02); had higher C-reactive protein (10.48 ± 7.19 vs. 6.03 ± 10.48 mg/dL, *P* < 0.0001) and D-dimer (2.38 ± 3.42 vs. 0.81 ± 0.59 μg/mL, *P* < 0.0001) at admission; and if hospitalized, had a longer length of stay (10.36 ± 10.85 vs. 3.0 ± 1.73 days, *P* < 0.0001) (Table 1). None of the pediatric patients required oxygen, whereas 7 adults did and 4 required mechanical ventilation (*P* = 0.03). Four adults but none of the pediatric patients died.

To evaluate whether the age-related differences in outcomes reflect differences in host responses at the site of viral exposure, the nasal mucosa, we performed RNA-Seq on cells isolated from NP swabs obtained at the time of presentation to the emergency department (*n* = 6 pediatric and 15 adult patients). The expression of RPLP0, a housekeeping gene, was similar in the pediatric (mean counts = 2667 ± 900.9) and adult (3144 ± 1961) samples. Expression of ACE2 trended toward being higher in the pediatric samples (*P* = 0.053) and there were no significant differences in TMPRSS2 (protease that cleaves the spike protein into its 2 active components, S1 and S2) expression (Figure 1B). Overall, we identified 538 differentially expressed genes (*P* < 0.05 after FDR correction), including 267 that were upregulated and 271 that were downregulated when comparing pediatric and adult patients (Figure 1C).

Principal component analysis (PCA) of the RNA-Seq data set separated pediatric and adult patients into nonoverlapping clusters, with the first dimension capturing 18.4% of the overall variance, the second dimension capturing 12.9%, and the third 7.9% (Figure 1D). To understand the gene expression patterns contributing to the segregation of the samples, we interrogated the 50 genes contributing most strongly to the principal components (Supplemental Table 1; supplemental material available online with this article; https://doi.org/10.1172/jci.insight.148694DS1). This gene set was enriched for IFN-stimulated and other innate immune response genes such as *IL1B*, *CCL3*, *CXCL10*, and *NLRP3* (Figure 1E). After dichotomization of adults based on requirement for respiratory support, PCA analysis showed that adults who never required supplemental oxygen were in an intermediate category compared with adults who required supplemental oxygen and children (Supplemental Figure 1).

**Figure 2:**
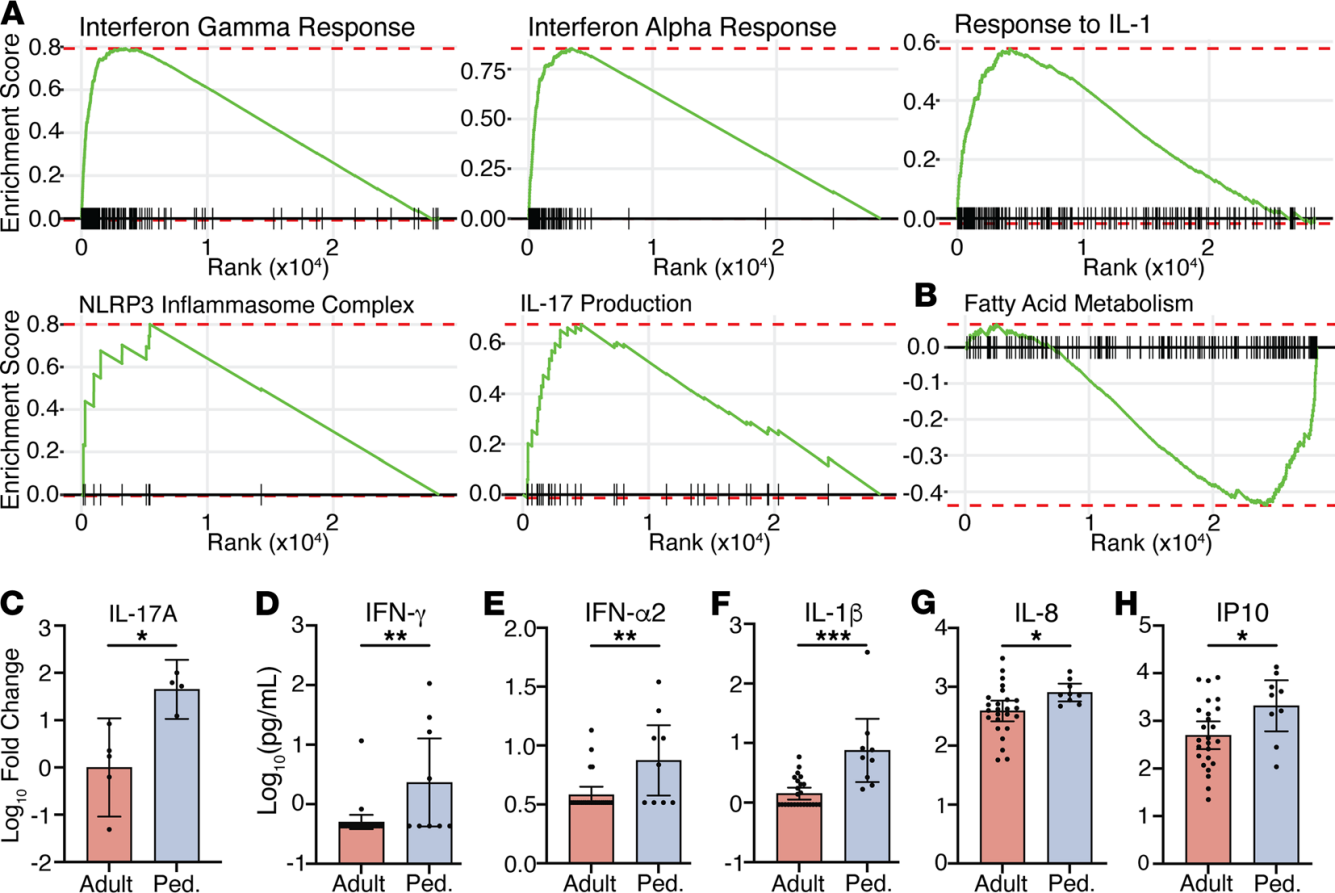
Innate responses in pediatric and adult nasopharynx. (**A**) Gene set enrichment plots for the indicated pathways: IFN-γ response (normalized enrichment score [NES] = 3.66, *P* = 0.006), IFN-α response (NES = 3.52, *P* = 0.006), IL-1 response (NES = 2.70, *P* = 0.03), NLRP3 inflammasome (NES = 1.88, *P* = 0.03), and IL-17 production (NES = 2.30, *P* = 0.03). (**B**) Fatty acid metabolism (NES = –1.86, *P* = 0.006). All plots show enrichment in RNA-Seq data from pediatric patients relative to adult patients. (**C**) Relative IL-17A gene expression measured by RT-qPCR in 5 adult and 4 pediatric samples not used for RNA-Seq. Fold change was calculated by the 2^CT(ref)–CT^ method using the mean adult value as the reference. (**D**–**H**) Levels of the indicated cytokines in NP transport media from 25 adult and 9 pediatric patients measured by multiplex Luminex assay. *P* values are listed above comparison bars. Unpaired *t* test (**G** and **H**) or Mann-Whitney test (**C**–**F**). Bars show mean ± 95% CI. *P < 0.05; **P < 0.01; ***P < 0.001. Ped., pediatric.

Given the strong contribution of innate immune gene expression in our PCA, we performed Gene Set Enrichment Analysis (GSEA) to evaluate the immune landscape. This showed enrichment of genes in the IFN-γ response (hallmark: M5913, normalized enrichment score [NES] = 3.66, *P* = 0.006), IFN-α response (hallmark: M5911, NES = 3.52, *P* = 0.006), IL-1 response (GO: 0070555, NES = 2.70, *P* = 0.03), NLRP3 inflammasome (GO: 0072559, NES = 1.88, *P* = 0.03), and IL-17 production (GO: 0032620, NES = 2.30, *P* = 0.03) in pediatric relative to adult patients (Figure 2A). Conversely, fatty acid metabolism (hallmark: M5935, NES = –1.86, *P* = 0.006) was enriched in adults compared with pediatric patients (Figure 2B). Oxidative phosphorylation and glycolysis pathways were also increased in adults compared with children, but these were not statistically different after FDR correction (NES = –1.18, *P* = 0.14; NES = –1.09, *P* = 0.3, respectively). These findings further support an enhanced innate response to SARS-CoV-2 infection in children but enhanced metabolic pathways among cells from adults.

We then performed quantitative reverse-transcription PCR (RT-qPCR) using RNA isolated from NP swabs from an additional 4 pediatric and 5 adult patients who were not included in the RNA-Seq analysis to confirm these findings. IL-17A gene expression was significantly increased in children (Figure 2C), and there were similar trends for higher levels of IFN response genes (Supplemental Figure 2). We also measured cytokine levels in NP fluid and found significantly higher levels of IFN-γ, IFN-α2, and IL-1β, IL-8, and IP10 in pediatric patients (Figure 2, D–H), consistent with the RNA-Seq data. IL-17A protein levels also trended toward a higher value in pediatric versus adult patients, but the measured values were all less than 10 pg/mL, which is near the lower limit of detection of the assay.

**Figure 3:**
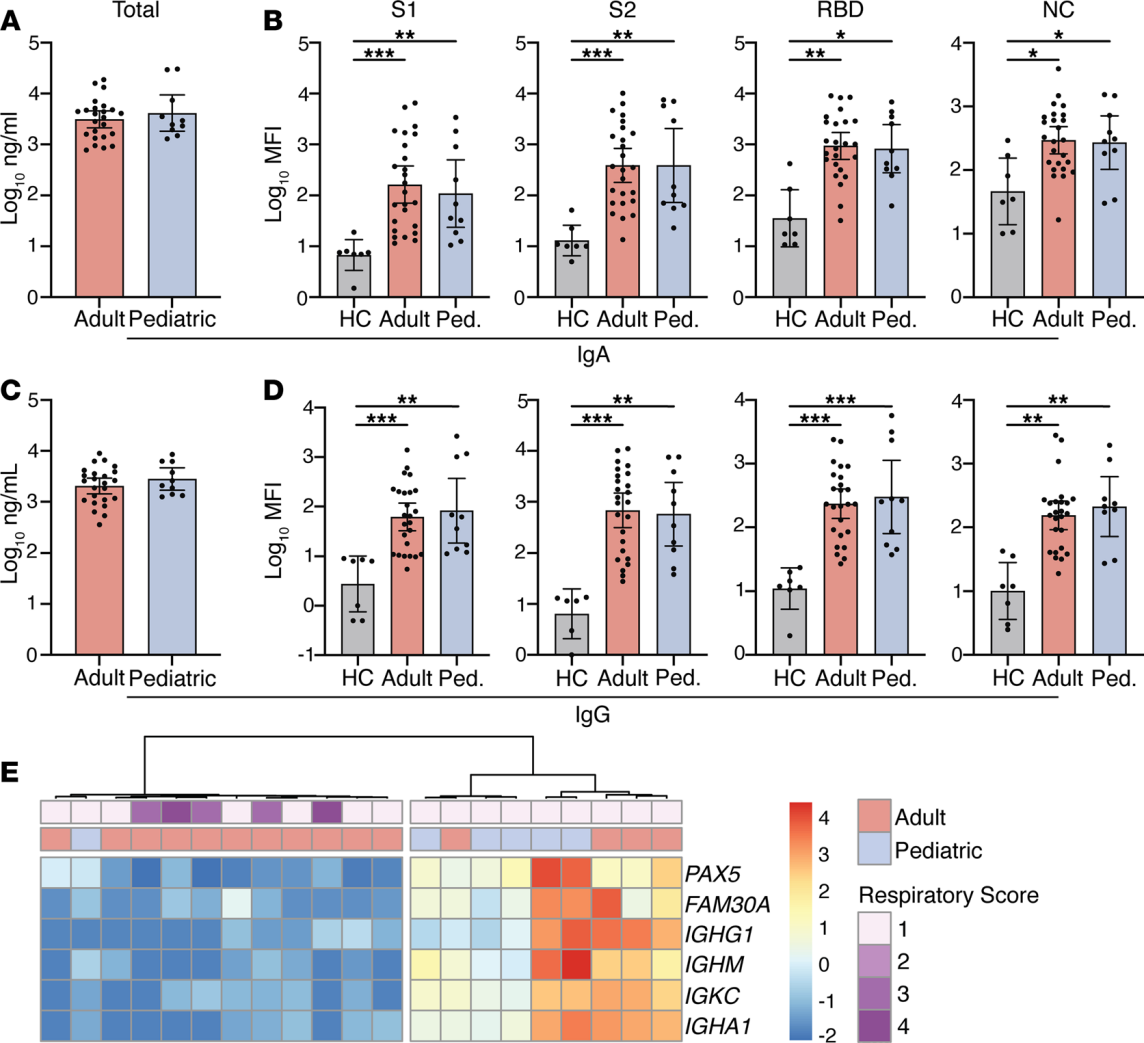
Early mucosal antibody responses in pediatric and adult COVID-19 patients. (**A**) Total and (**B**) SARS-CoV-2–specific IgA and (**C**) total and (**D**) SARS-CoV-2–specific IgG levels at time of presentation were measured in 10 pediatric and 25 adult patients and 7 healthy controls (HCs). (**E**) Heatmap showing expression of B cell–related genes contributing to PC1-3. Annotations show age group and peak respiratory score (1 = room air, 2–4 = supplemental oxygen). Total antibody levels (**A** and **C**) measured by ELISA; SARS-CoV-2 antibody levels (**B** and **D**) measured by multiplexed Luminex assay. Where significant, *P* values are listed above comparison bars; Kruskal-Wallis test (**B** and **D**). Bars show mean ± 95% CI. *P < 0.05; **P < 0.01; ***P < 0.001. Ped., pediatric.

SARS-CoV-2–specific IgA in nasal mucosal secretions may contribute to protection (9), and our RNA-Seq data revealed a cluster of B cell–associated genes in the PCA gene set (Dim 2; *IGHA1, IGKC, IGHM, IGHG1, FAM30A, and PAX5*) with higher expression in a subset of adult and pediatric samples (Figure 1E). Therefore, we measured total IgA and IgG by ELISA and SARS-CoV-2–specific IgA and IgG targeting S1, S2, and the receptor binding domain (RBD) of the spike and nucleocapsid (NC) protein by multiplex assay in the NP fluid. We did not identify differences in total IgG or IgA when comparing pediatric and adult patients (Figure 3, A and C). Although patients with COVID-19 had elevated levels of SARS-CoV-2–specific immunoglobulins compared with healthy controls (*n* = 7 adults), there were no significant differences between age groups (Figure 3, B and D). Agglomerative hierarchical clustering using only the B cell–associated genes from the RNA-Seq analysis dichotomized the cohort into a low or high expression group (Figure 3E). All of the adult patients with more severe clinical disease who required supplemental oxygen were in the low transcript group (Figure 3E).

**Figure 4:**
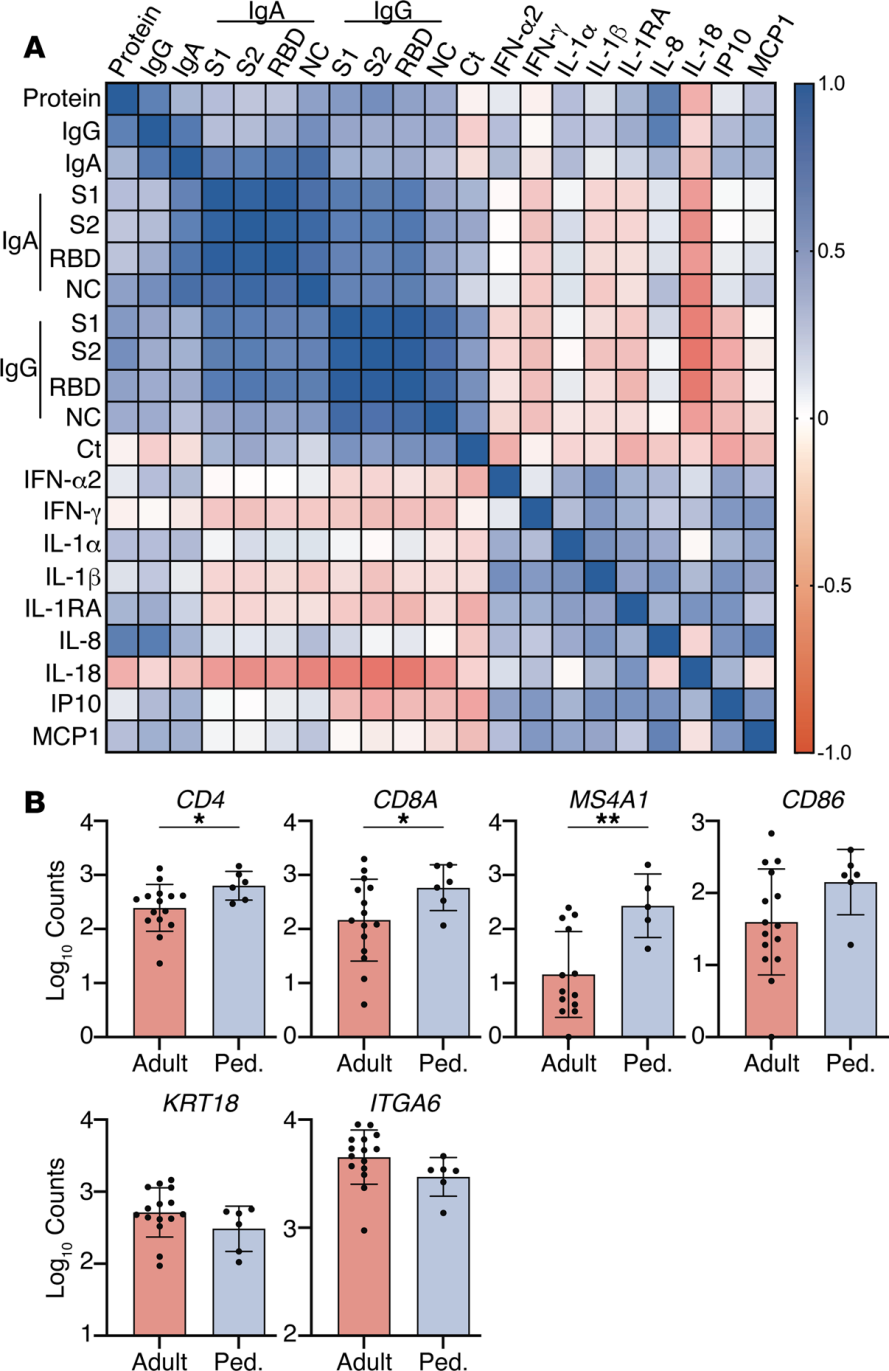
Associations between mucosal proteins and expression of immune or epithelial cell markers. (**A**) Spearman correlation matrix of Ct values, total protein recovered (NanoDrop), antibody, and cytokine protein levels in nasopharyngeal fluid. (**B**) RNA-Seq read counts for indicated genes in pediatric and adult patients *MS4A1* (CD20) (*P* = 0.004), *CD4* (*P* = 0.046), *CD8a* (*P* = 0.036), *CD86* (*P* = 0.054), *KRT18* (*P* = 0.084), *ITGA6* (*P* = 0.18); *n* = 15 adults and *n* = 6 pediatric patients; unpaired *t* test with Welch’s correction. Bars show mean ± 95% CI. *P < 0.05; **P < 0.01.

Correlation matrices were generated to explore associations between total protein concentration in NP swab transport media (measured by NanoDrop), antibody levels, cytokine levels, and SARS-CoV-2 Ct values (Figure 4A). In general, total protein correlated with antibodies but not with cytokines, and the antibodies and the cytokines did not strongly correlate with each other except for IL-18. There was a strong inverse correlation between IL-18 and anti–SARS-CoV-2 IgG (anti-S1 *r* = –0.63, *P* < 0.001; anti-S2, *r* = –0.67, *P* < 0.001; anti-RBD, *r* = –0.65, *P* = 0.006; and anti-NC, *r* = –0.48, *P* = 0.006) and IgA (anti-S1 *r* = –0.49, *P* = 0.004; anti-S2, *r* = –0.55, *P* = 0.001; anti-RBD, *r* = –0.50, *P* = 0.003; and anti-NC, *r* = –0.60, *P* < 0.001). The IL-18 levels were lower in adults who did or did not require supplemental oxygen (*P* = 0.12), but there were no significant differences in IL-18 levels when comparing children and adults.

Because the patterns of gene expression could reflect differences in cellular composition, we also compared expression of markers representing different cell populations in an exploratory analysis. The read counts for expression of genes representing immune cells were generally higher in children: *MS4A1* (CD20) (*P* = 0.004) as well as *CD4* (*P* = 0.046), *CD8a* (*P* = 0.036), and *CD86* (*P* = 0.054), whereas those associated with epithelial cells, including *KRT18* (Keratin 18) (10) and *ITGA6* (integrin alpha 6) did not differ in children versus adults (Figure 4B).

## Discussion

Our studies identified age-related differences in primary immune responses to SARS-CoV-2 at the nasal mucosa, the presumptive site of first viral encounter, which may contribute to the clinical outcomes. Most studies focus on systemic immune responses and have measured cytokines and antibodies in the blood, but our prior and other studies led us to speculate that the innate immune response was more vigorous in children (7). Our new findings using NP swabs obtained at the earliest time point of presentation established this and suggest that a more robust mucosal response in children overcomes viral evasion strategies and generates an immediate barrier to viral infection. This may dampen the subsequent adaptive systemic immune response, as evidenced by our prior findings of lower neutralizing antibodies, decreased antibody-dependent cell-mediated phagocytic activity, and less robust T cell responses in children versus adults (7). Reduced adaptive responses in children compared with adults in the peripheral blood has been confirmed by others who also documented decreased neutralizing antibody titers (11) and less robust T cell responses (12).

In contrast, a reduced or delayed mucosal response in adults, evidenced by the decreased expression of transcripts associated with innate pathways in their NP swabs, may lead to an inability to escape viral immune evasion and a more vigorous adaptive response (13, 14). The latter contributes to high systemic levels of other inflammatory cytokines (e.g., IL-6 and TNF) and an increased risk of acute respiratory distress syndrome, which was also observed with SARS-CoV-1 (15–18). Other studies have found an impaired innate response to SARS-CoV-2 in adults compared with their response to other respiratory viral infections (19).

Children have more frequent respiratory infections than adults. This, as well as recent childhood immunizations, could contribute to a higher basal level of activation of mucosal immune responses, as suggested by studies of rhinovirus in which an early IFN response was associated with rapid viral clearance (20). Notably, a recent study found that infection of organoid cultures with rhinovirus protected against subsequent SARS-CoV-2 challenge (21). The prior respiratory infections in children may prime immune cells that can rapidly respond to SARS-CoV-2. The higher gene expression of metabolic flux pathways in adults may reflect the metabolic demands needed to activate innate pathways (22) and may account for a delayed response with COVID-19 (23).

We detected similar levels of SARS-CoV-2–specific IgA and IgG in NP samples obtained from adults and children at this early time point before a fully mature antibody response would be expected. Notably, these early antibody responses correlated negatively with mucosal IL-18 levels. IL-18, an IL-1 superfamily cytokine predominantly produced by macrophages, is cleaved to its active form by the NLRP3 inflammasome and ultimately promotes the production of IFN-γ. We speculate that the early release of this cytokine may temper the adaptive response, which is consistent with the more severe outcomes with lower levels of IL-18. The kinetics of its secretion, however, may be important because elevated serum IL-18 levels later in the disease course are associated with increased inflammasome activation and disease severity (24).

Our data suggesting increased expression of genes that are markers of immune cells including B and T cells and possibly antigen-presenting cells in NP swabs obtained from pediatric compared with adult patients is consistent with an enhanced immune response. The increase in CD20 expression as well as the inverse relationships between Ig gene expression and clinical outcomes suggest that a robust B cell response in the nasopharynx is protective. However, direct studies of the B cells from this site are needed to support this notion.

There are several limitations to this analysis, including the relatively small sample size. Variability in NP swab technique could have contributed to observed differences in gene expression and protein levels, although expression of RPLP0 and nasal fluid total protein were similar in children and adults. The levels of IgG and IgA correlated with total protein recovered, but a more comprehensive proteome analysis may identify other compositional differences that contribute to age-associated responses to SARS-CoV-2. We did not directly examine the cellular composition of the NP swabs except with an exploratory analysis of the RNA-Seq data. It is possible that with a larger sample size, we would be able to identify additional differences in cellular markers that contribute to our findings. An analysis of gene expression on a per-cell basis would have also enhanced our analysis since we cannot be certain whether our findings reflect differences in cellular composition or gene expression by individual cells.

In summary, we showed, for the first time to our knowledge, direct evidence for a more vigorous early immune response and activation of innate immune pathways in the nasopharynx of children compared with adults at clinical presentation with COVID-19. Innate responses to other pathogens have also been shown to decrease with age (25, 26). The cellular source of these protective cytokines is not clear but could include mucosal and airway epithelial cells, invariant natural killer T cells, or other immune cells (27, 28). Regardless of the source, our findings suggest that airway-resident cells may establish the response to the virus that ultimately determines the clinical outcomes. The reasons for the age-associated differences in the early immune responses to SARS-CoV-2 require further study, but therapies that enhance these pathways may be an effective treatment strategy and help protect patients from severe outcomes, as suggested by ongoing trials of inhaled IFN-β1 for early treatment of COVID-19 (29).

## Methods

### Study design.

NP swabs were obtained from the Clinical Microbiology Laboratory at Montefiore Medical Center from pediatric (younger than 18) and adult patients with confirmed SARS-CoV-2 infection by PCR assay who presented to the emergency department at Montefiore Medical Center between November 2020 and January 2021. Patients were excluded if they had preexisting medical conditions that might affect immune responses, including cancer and HIV; were pregnant; or were receiving chronic immunosuppressive therapy. Demographics; length of stay; peak respiratory support required (1 = room air, 2 = nasal cannula, 3 = CPAP or high-flow nasal oxygen, and 4 = mechanical ventilation); outcome; and the results of clinical laboratory studies, including SARS-CoV-2 PCR (Ct values), complete blood counts, D-dimer, and C-reactive protein were obtained by chart review.

The NP swabs were collected in viral transport medium. A portion was removed for measurement of SARS-CoV-2 RNA by PCR in the Clinical Microbiology Laboratory at Montefiore Medical Center and the remainder of the sample was transported. NP swabs were also obtained from 7 healthy young adult controls. The swab was transferred to a tube containing media supplemented with 13 μM dithiothreitol to inactivate virus and then incubated in a thermomixer for 10 minutes at 37°C and 500*g*. The cells were washed and frozen in 500 μL 90% FBS/10% DMSO. The original transport medium (nasal fluid) was aliquoted and stored at –80°C for measurement of cytokines and antibodies.

### RNA-Seq.

RNA was isolated from cryopreserved cells using the miRNeasy Micro kit (QIAGEN, 217084). Samples with sufficient RNA quantity and quality were used for analysis. Libraries were prepared at the Yale Center for Genome Analysis using the NEBNext rRNA Depletion Kit (New England Biolabs E6310L). Individual samples or pools from 2 patients of similar age and outcome (*n* = 4 pools, adults) were normalized to 1.2 nM and loaded on an Illumina NovaSeq S4 flow cell to generate 30 M read-pairs per sample. Samples were checked for read quality and adapter contamination using FastQC and aligned to transcripts using the GENCODE transcript sequences (version 33) as the reference file with Salmon (30). All analyses in R were performed using R version 4.0.3. Transcripts were mapped to genes using tximport. Differential gene expression analysis was performed with DESeq2 (31). Heatmaps were generated using the pheatmap package. PCA was performed with the prcomp function using all genes with a nonzero total read count. Prior to PCA, data were transformed with the vst function in DESeq2. PCA results were visualized with the factoextra package. For GSEA, Hallmark (h) and GO (c5.go) data sets were downloaded from MSigDB (Broad Institute) and analysis performed in R with the fgsea package using 1000 permutations.

### Real-time RT-qPCR.

qPCR was performed using the TaqMan Gene Expression Master Mix (Applied Biosystems, Thermo Fisher Scientific, 4369016). Data were analyzed by the 2-DDCt method using the mean adult values as the reference. Primers/probes were from Thermo Fisher Scientific: MX1 (Hs00895608_m1), MX2 (Hs01550811_m1), IFNA1 (Hs00256882_s1), IFI44 (Hs00951349_m1), IFIT1 (Hs03027069_s1), IL17A (Hs00174383_m1), IFNG (Hs00989291_m1), RPLPO (4326314E).

### Cytokine and antibody measurements.

Media from NP swabs were thawed and treated with UV light to inactivate virus prior to use. SARS-CoV-2–specific IgG and IgA were measured using MILLIPLEX SARS-CoV-2 Antigen Panel 1 IgG and IgA kits (MilliporeSigma, HC19SERG1-85K and HC19SERA1-85K, respectively). Cytokines were measured using the MILLIPLEX Human Cytokine/Chemokine/Growth Factor Panel A kit (MilliporeSigma, HCYTA-60K). Data from Luminex assays were acquired on a Luminex MAGPIX and analyzed in the MILLIPLEX Analyst program (MilliporeSigma). Total IgA was measured using the IgA Human ELISA Kit (Invitrogen, Thermo Fisher Scientific, BMS2096) and total IgG measured using the IgG Human ELISA Kit (Invitrogen, Thermo Fisher Scientific, BMS2091). ELISA data were acquired on a Spectra Max M5 using SoftMax Pro 7.1 GxP software (both Molecular Devices). Total protein was measured by NanoDrop.

### Statistics.

Because of sample volume limitations, not all assays could be performed with all samples. Missing data were at random and the number of samples used is indicated. Statistical analyses were performed in GraphPad Prism (version 9.0.1). All *t* tests were 2 tailed. Cytokine and antibody data were log-transformed prior to analysis. Normality was tested and a parametric or nonparametric test was used for comparison of groups as indicated. For the exploratory analysis of the expression of specific genes, we did not correct for multiple comparisons. *P* values less than 0.05 were considered significant.

### Study approval.

This study was approved by the IRB of the Albert Einstein College of Medicine (IRB 2020-11278). Written informed consent was obtained for samples from healthy controls.

## Author contributions

CAP, KCH, and BCH designed the study; CAP performed experiments; BG, SS, DYG, EO, and MJK performed data acquisition and analysis; CAP, MJK, BCH, and KCH wrote and edited the manuscript.

## Supplementary Material

Supplemental data

Trial reporting checklists

ICMJE disclosure forms

## Figures and Tables

**Table 1:**
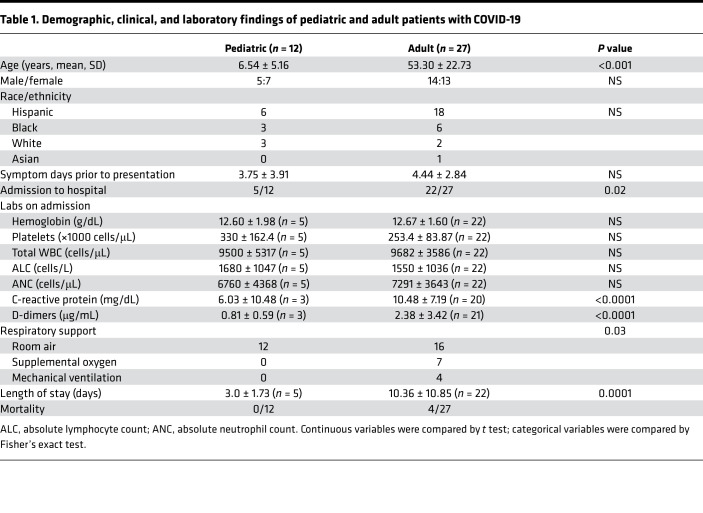
Demographic, clinical, and laboratory findings of pediatric and adult patients with COVID-19

## Version 1 04/06/2021

In-Press Preview

## Version 2 05/10/2021

Electronic publication
