# Supplementary material for: Natural mucosal barriers and COVID-19 in children
Source: JCI Insight. 2021 May 10;6(9):e148694. doi: 10.1172/jci.insight.148694 (PMC8262299; doi:10.1172/jci.insight.148694)
Supplement: Supplemental data [file jciinsight-6-148694-s054.pdf]

Supplementary Information

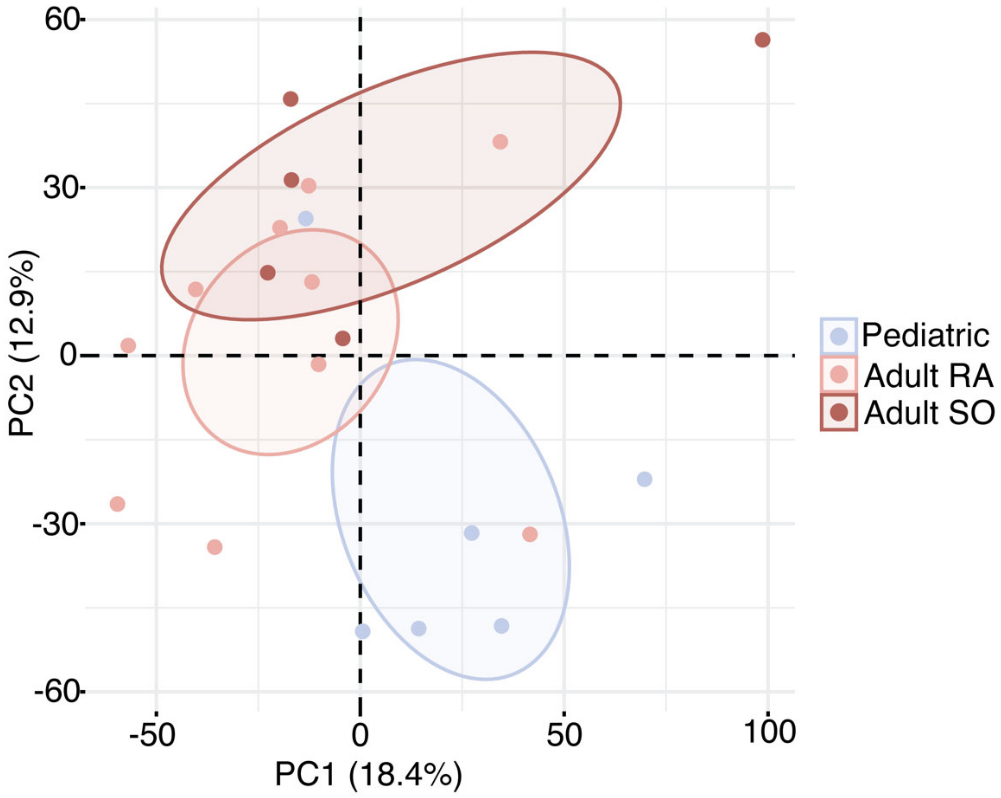

Figure S1: Adults not requiring oxygen are intermediate between children and adults who did. Principal component plot showing 95% confidence ellipses for pediatric samples, and samples from adults who did or did not require supplemental oxygen. RA = Room Air; SO = Supplemental Oxygen; N = 6 pediatric, 10 RA, 5 SO.

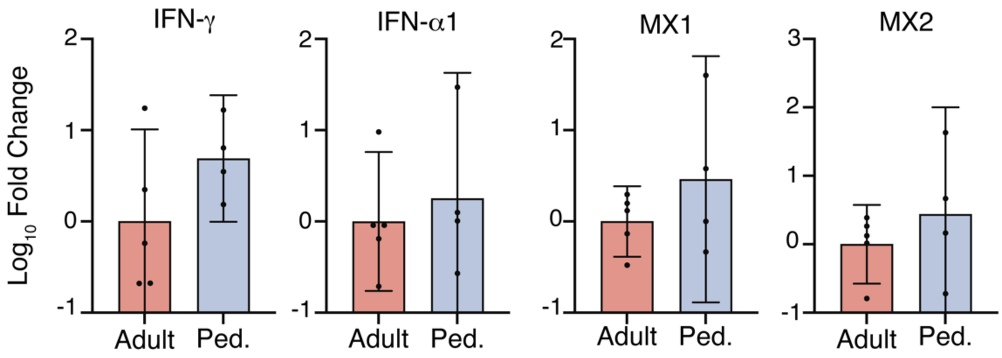

Figure S2: RT-qPCR of interferon-related genes. RT-qPCR was performed on indicated genes using 5 adult and 4 pediatric samples not included in the RNAseq analysis. Bars show mean ± 95% CI. Ped. = Pediatric. Fold change was calculated by the $2^{\Delta\Delta Ct}$ method using the mean adult value as the reference.

Supplemental Table 1: 50 top contributing genes to Principal Component (PC) 1, PC2, and PC3 and their relative contributions.

PC1 (18.4%)		PC2 (12.9%)		PC3 (7.9%)	
**Gene**	**Contribution**	**Gene**	**Contribution**	**Gene**	**Contribution**
TREM1	0.173227469	CXCL10	0.261895036	IFIT2	0.22523612
IL1B	0.173026738	GNLY	0.196726134	ISG15	0.2089935
NLRP3	0.172496561	CXCL11	0.151920142	IFI44L	0.202055232
PLEK	0.162704966	HLA-DQA1	0.146738606	IDO1	0.200463613
ITGAX	0.157733948	IGHM	0.146005498	IFIT3	0.195493576
G0S2	0.154580633	IL4I1	0.142580142	OASL	0.18404088
AQP9	0.148861898	IL2RB	0.140651881	CXCL10	0.183737491
CSF3R	0.148636646	PAX5	0.138820826	IFIT1	0.178488805
DYSF	0.136463767	GZMB	0.138511873	DUOXA2	0.175129846
CD93	0.135889286	PRF1	0.137850844	COL6A1	0.165063694
PLAUR	0.132820742	RGS1	0.13718506	HERC6	0.151114668
SLC7A5	0.128115574	MUC13	0.136991266	RN7SK	0.149871263
LCP2	0.125730173	CCL4	0.13562182	DDX58	0.146155476
PDE4B	0.120612726	SAMD9L	0.13544711	XAF1	0.144283934
SRGN	0.120542836	ISG15	0.13181772	OAS2	0.140052543
SLA	0.119850865	CALHM6	0.130468506	DUOX2	0.137060231
HCK	0.114471549	IFIT2	0.124153109	LGALS17A	0.136753726
SPP1	0.113627752	GBP4	0.123725266	GBP4	0.132043581
CXCL8	0.113298653	CCL8	0.12346328	LIF	0.130461286
OSM	0.112279813	SERPINA3	0.114540728	SAMD9L	0.128121837
BCL2A1	0.111565919	FAM107A	0.11310861	OAS3	0.118920822
SPI1	0.111537278	CD69	0.11108973	ITGAM	0.118378247
SLC2A3	0.109418219	MUC2	0.110222241	SLC26A4	0.116611424
FGR	0.10911398	IGHA1	0.10912497	CMPK2	0.114789211
PIK3R5	0.108443558	GBP5	0.108825097	CXCL11	0.114306252
CSF2RB	0.107802479	ECM1	0.108101562	IFI44	0.114218194
CXCL5	0.107188795	IRF8	0.107731695	DDX60	0.113710772
IL1R2	0.107005515	FAM30A	0.107637	USP18	0.112839306
CYBB	0.106642518	CCR5	0.107139996	OAS1	0.109011191
ADGRG3	0.105716537	LILRB1	0.105429284	IFITM1	0.107813459
FCGR2A	0.105497597	TRIM31	0.104974062	WARS1	0.103784933
SAMSN1	0.103942468	SIGLEC1	0.102339331	IGHG1	0.102064625
LOC388813	0.103942468	CXCL9	0.101556335	PARP14	0.099055872
CCL3	0.102794696	MMP10	0.101522671	LRRN2	0.09855234
C17orf107	0.10140178	HLA-DOA	0.100648768	RSAD2	0.097553013
HCAR3	0.101354907	HLA-DPA1	0.100443991	BATF2	0.096559529
CD83	0.101054539	ACOD1	0.099951686	TNFSF10	0.095625082
ITGAM	0.100376293	CCL3	0.098545026	ATP10B	0.093752845
TNFRSF1B	0.099995775	CMKLR1	0.098249242	FCGBP	0.092020712
LCP1	0.099685455	IGKC	0.097192845	ETV7	0.089949386
INHBA	0.09935401	IFIT3	0.097086428	ISG20	0.089559704
MCEMP1	0.09747709	HSD11B2	0.096913486	AMY1C	0.089451592
PELATON	0.096975261	LGALS17A	0.096813097	MX1	0.087339886
ZEB2	0.096361645	KRT13	0.095294057	CXCL5	0.086400952
LAPTM5	0.095338525	THSD7A	0.094944543	MKI67	0.085157719
FFAR2	0.095280218	SERPING1	0.090751263	MX2	0.084751699
CCL3L1	0.094992222	MTCO1P12	0.090364564	CES1P1	0.084620747
CCL3L3	0.094992222	ANPEP	0.089523297	MMP9	0.084573643
LINC00528	0.092864705	IFIT1	0.088527915	TRIM31	0.084563359
ALOX5AP	0.091639754	ZAP70	0.087572246	IFI6	0.081838042
